# Improving neuroendocrine tumor treatments with mathematical modeling: lessons from other endocrine cancers

**DOI:** 10.1530/EO-24-0025

**Published:** 2025-02-05

**Authors:** John Metzcar, Rachael Guenter, Yafei Wang, Kimberly M Baker, Kate E Lines

**Affiliations:** ^1^ Department of Intelligent Systems Engineering, Luddy School of Informatics, Computing and Engineering, Indiana University, Bloomington, Indiana, USA; ^2^ Department of Informatics, Luddy School of Informatics, Computing and Engineering, Indiana University, Bloomington, Indiana, USA; ^3^ Therapy Modeling and Development Center, University of Minnesota-Twin Cities, Minneapolis, Minnesota, USA; ^4^ Department of Surgery, Heersink School of Medicine, University of Alabama at Birmingham, Birmingham, Alabama, USA; ^5^ Department of Biology, Shaheen College of Arts and Sciences, University of Indianapolis, Indianapolis, Indiana, USA; ^6^ OCDEM, Radcliffe Department of Medicine, University of Oxford, Churchill Hospital, Oxford, UK; ^7^ Department of Medical and Biological Sciences, Oxford Brookes University, Oxford, UK

**Keywords:** neuroendocrine tumors, systems biology, multiple neuroendocrine neoplasia, virtual clinical trials, clinical mathematical oncology modeling

## Abstract

Neuroendocrine tumors (NETs) occur sporadically or as part of rare endocrine tumor syndromes (RETSs) such as multiple endocrine neoplasia 1 and von Hippel–Lindau syndromes. Due to their relative rarity and lack of model systems, NETs and RETSs are difficult to study, hindering advancements in therapeutic development. Causal or mechanistic mathematical modeling is widely deployed in disease areas such as breast and prostate cancers, aiding the understanding of observations and streamlining *in vitro* and *in vivo* modeling efforts. Mathematical modeling, while not yet widely utilized in NET research, offers an opportunity to accelerate NET research and therapy development. To illustrate this, we highlight examples of how mathematical modeling associated with more common endocrine cancers has been successfully used in the preclinical, translational and clinical settings. We also provide a scope of the limited work that has been done in NETs and map how these techniques can be utilized in NET research to address specific outstanding challenges in the field. Finally, we include practical details such as hardware and data requirements, present advantages and disadvantages of various mathematical modeling approaches and discuss challenges of using mathematical modeling. Through a cross-disciplinary approach, we believe that many currently difficult problems can be made more tractable by applying mathematical modeling and that the field of rare diseases in endocrine oncology is well poised to take advantage of these techniques.

## Introduction

Neuroendocrine tumors (NETs) develop from the diffuse neuroendocrine system and arise throughout the body, including in the gastrointestinal tract, pancreas, lungs and thyroid. They can occur as sporadic tumors or as part of inherited rare endocrine tumor syndromes (RETSs), such as multiple endocrine neoplasia type 1 (MEN1). The incidence of NETs is rising globally, with an estimated 8.8 per 100,000 people diagnosed in England and approximately 10 per 100,000 people diagnosed in the United States (Chauhan *et al.* 2020, Das & Dasari 2021, White *et al.* 2022). Some NETs are functional, meaning they are hormonally active. Patients may experience debilitating symptoms due to secretion of excess neuropeptides and hormones from the tumors. These symptoms vary depending on the released hormone and include diarrhea, wheezing, carcinoid heart disease, flushing and skin rashes (Rindi *et al.* 2022, Vitale *et al.* 2023). Surgical resection is the only potentially curative option for patients; however, 20–50% of patients present with disease unsuitable for complete resection (Roeyen *et al.* 2009, Riihimäki *et al.* 2016, Krug *et al.* 2022). First-line therapy constitutes the use of somatostatin analogs, which help alleviate symptoms and can prolong progression-free survival (O’Dorisio *et al.* 2020). Other treatment options include peptide receptor radionuclide therapy (PRRT), everolimus (mTOR inhibitor), sunitinib (tyrosine kinase inhibitor), chemotherapy or liver-directed therapies such as ablation or chemoembolization (Zandee & de Herder 2018, O’Dorisio *et al.* 2020). Given the current therapeutic landscape, patients with localized disease have a 5-year survival rate ranging from 78 to 93%, but this rate drops to 19–38% for patients with metastatic disease (Riihimäki *et al.* 2016). This difference in outcomes indicates the urgent need for new evidence-based treatments to extend patient survival and improve disease management in the metastatic setting. Furthermore, in RETSs, which can include multiple primary tumors developing across time, lifelong strategies are needed.

Compared to many other cancers, scientific research is relatively lacking in NETs, with several factors likely contributing to this (Chauhan *et al.* 2020, Herring *et al.* 2021, Sedlack *et al.* 2022). First, NETs are less common and receive limited attention and awareness compared to other cancers, resulting in relatively lower research funds (Chauhan *et al.* 2020). Second, NETs occur in multiple different organs, including the pancreas, lungs and small bowel, sometimes simultaneously, and therefore represent a diverse range of pathologies. Third, available preclinical models are limited (April-Monn *et al.* 2020, Chauhan *et al.* 2020, Herring *et al.* 2021, Sedlack *et al.* 2022, Viol *et al.* 2022). Relatively few immortalized NET cell lines exist, and they only partially reflect the pathophysiology of NETs observed clinically. There has been success in 3D spheroid model development for NETs, but more options are needed to reflect the heterogeneity of NETs (Ear *et al.* 2019, Forsythe *et al.* 2023, April-Monn *et al.* 2024). Furthermore, there are no widely available patient-derived xenograft nor genetically engineered animal models. Finally, the genetics and epigenetics underlying the development of NETs, which affect both treatment outcomes and prognosis, are diverse. For example, more than 20 genes are currently implicated in pancreatic NETs alone (Di Domenico *et al.* 2017, Scarpa *et al.* 2017). A lack of research funding and optimal preclinical models combined with diverse, complicated biology hampers the understanding of NET biology, drug development and clinical trials, particularly in the RETSs (April-Monn *et al.* 2020, Chauhan *et al.* 2020, Herring *et al.* 2021, Sedlack *et al.* 2022, Viol *et al.* 2022).

To address this, we propose taking advantage of mechanistic mathematical modeling to complement currently utilized research techniques for NETs. Mathematical modeling, which can synthesize existing observations and data and aid in planning additional experiments, is already being applied to aid in exploring, understanding and treating other cancers and provides invaluable insights into experimental design, optimal treatment schedules and patient-specific responses to interventions. In this review, we focus on computational systems biology, pharmacological modeling, *in silico* virtual clinical trials and clinical mathematical oncology. We present examples from more common endocrine cancers (prostate and breast) to highlight successes using these techniques, scope out the limited literature available in mathematical modeling of NETs, describe practical aspects of the techniques and finally map how modeling might be used in neuroendocrine tumors to improve NET treatment discovery and patient outcomes. In doing so, this review offers new perspectives and research avenues for using mathematical modeling, paving the way for additional efforts that will in turn spurn the acquisition of new knowledge in the diagnostics and therapeutics in NETs.

## Mathematical and computational modeling

**Figure 1:**
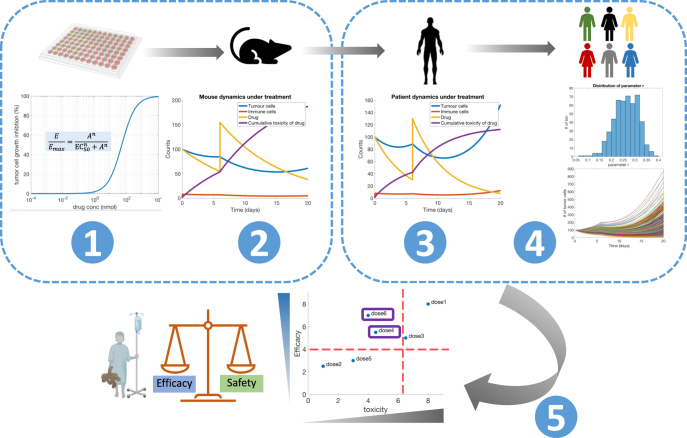
Mathematical modeling throughout the translational spectrum: mathematical and computational models integrate observations, for example data generated from cell lines (1), data from *in vivo* mouse studies (2) or patient data (3, 4, 5) to provide quantitative descriptions or predictions that are not possible with each dataset alone. Systems biology modeling can aid in discovery (1) and preclinical (2) stages. Pharmacokinetics and pharmacodynamics modeling explore dose exposure–response relationships and are standard in predicting dose for first-in-human studies (3). Their extensions quantitative systems pharmacology and physiologically based pharmacokinetics also inform translational science being used in the preclinical, translational and clinical spaces. Virtual clinical trials help to understand variability in responses and biomarkers for trials, making use of both pharmacokinetics and systems approaches (4). Finally, clinical mathematical modeling approaches can combine modeling of disease processes with patient biomarkers and outcomes to both optimize treatment approach and predict efficacy and safety of treatment regimens. Example model outputs generated from MATLAB simulation code (with slight adaptations), were originally made available through the *Centre de recherches mathematiques (CRM) Computational Modeling of Cancer Biology and Treatments Workshop. *Original code available at https://github.com/mlcraig/ComputationalBiologyFittingTutorial. Figure created in part with BioRender.com.

Biological mathematical modeling is a technique that incorporates mechanistic or causal links between dynamic variables using equations or other mathematical objects to represent the behavior of biological systems. These representations are encoded and then simulated in a computational system, forming a computational model. This technique can combine large datasets and synthesize diverse observations generated from a range of experiments and patient data (Fig. 1) to explore and explain complex phenomena (Clarke *et al.* 2019, McDonald *et al.* 2023). Mathematical and computational models integrate and inform observations by reproducing aspects of complex biological systems, allowing researchers to define the important variables in a process, reason through the limits of the system and ultimately enable quantitative descriptions and predictions unattainable through experiments alone (Leonard-Duke *et al.* 2020, McDonald *et al.* 2023). Furthermore, to enable predictions and hypothesis testing on individuals and populations, modelers seek to match the behavior of mechanistic models to data (both preclinical and clinical). Modeling aids in designing experiments, optimizing treatment schedules and predicting patient-specific responses (McDonald *et al.* 2023). Systems biology and multiscale modeling, pharmacological modeling, virtual clinical trials and clinical mathematical oncology are all paradigms within mechanistic modeling. We present each paradigm followed by examples from breast and prostate cancer that demonstrate the potential of mathematical modeling. We define key terms of mathematical modeling in Table 1, and a summary of these paradigms, including advantages and disadvantages, can be found in Table 2.

**Table 1:** Definitions of key mathematical modeling terms and techniques.

Term/technique	Definition
Boolean network	A network representing interacting biochemical species and processes, with each variable updated based on Boolean logic
Computational model	An encoded mathematical model that can be used to investigate dynamic biological systems and be able to be calibrated to datasets and predict specific scenarios
Flux balance analysis	A method for representing flow through a metabolic network, given physical and biological constraints
Mathematical modeling	Technique for representing dynamic biological systems using equations
Mechanism of action	Causal or molecular scale interactions that form the mechanisms by which a drug has an effect
Multiscale model	Mathematical model that spans two or more spatial or time scales, such as modeling cellular proliferation dynamics (subcellular) and cellular interactions
Ordinary differential equations (ODEs)	Equations that describe growth and decay of quantities based on dynamic rates of change
Pharmacodynamics (PD)	Biological effects of a compound (drug)
Pharmacokinetics (PK)	The dynamics of compound (drug) concentration in serum accounting for absorption, distribution, metabolism and excretion of the drug over time
Pharmacology modeling	Mathematical modeling related to drug development and safety
Physiologically based pharmacokinetics	Expanded pharmacokinetics model that accounts for organ-level details of drug absorption, distribution, metabolism and excretion
Quantitative systems pharmacology (QSP)	Mathematical modeling related to drug development in which a mechanism of action is described at a molecular level
Systems biology	Approach that views biochemical interactions as a set of interconnected pathways, allowing the study of a whole system rather than disconnected variables
Virtual clinical trial	Simulation of possible therapeutic regimens in a diverse virtual population

**Table 2:** Categories of modeling techniques used in translational research with mathematical examples and biological utility.

Modeling category	Mathematical technique and representative software frameworks/packages*	Example use cases	Example cases of biological utility	Advantages	Limitations	Examples of data used to develop and/or validate models
Systems biology	Boolean network (BoolNet (Müssel *et al.* 2010), MaBoSS (Stoll *et al.* 2017), cubewalkers (Park *et al.* 2023))	Gene regulation, signal transduction	Modeling cellular information processing such as the decision to commit to apoptosis	Requires only limited data: qualitative up/down responses	Challenging to capture continuous values such as concentration or relationships (like dose–response)	RNA expression data and/or phosphorylation data under conditions of interest
	ODEs (Python, MATLAB, R)	Metabolism, gene regulation, signal transduction	Simulating intracellular dynamics resulting in changes in biomarker production	Captures general behavior in a wide variety of systems; requires relatively little computing power; can be used for population fitting from molecule scale to clinical population	Time-dependent data are required to parameterize rates of change; presents major challenges for representing spatial biology and dynamics	Cell populations change over time, such as lymphocyte count or flow cytometry data; alterations in gene and protein expression
	Flux balance analysis (COBRA via MATLAB (Heirendt *et al.* 2019))	Metabolism	Representing production of energy within a cell	Can handle many reactions simultaneously	Assumes a steady state or no change in intracellular metabolites	Concentration of metabolites of interest under various environmental conditions
	Multiscale modeling (CompuCell 3D (Swat *et al.* 2012), PhysiCell (Ghaffarizadeh *et al.* 2018))	Cell–cell and cell–environmental interactions	Cell–cell signaling and heterogeneity of expression; cancer evolution; pressure-regulated tumor progression; hypoxia-driven tumor cell migration	Can represent microscopic phenomena, heterogeneity in disease development and complex mechanisms of actions such as physical and biochemical interactions in spatial scales	The multiple parameters in a multiscale model may be difficult to set with data, may require intensive computational resources and more specialized data and may require additional custom coding	Cell–cell interaction rates, microscopy images, spatial transcriptomics
Pharmacological	Varies – tends to be ODEs (Monolix, Phoenix, GastroPlus, Simbiology via MATLAB)	Distribution of chemicals in animals and drug development (PK) and their effects (PD)	Modeling drug concentration across cell, tissue, organs and whole body and investigation of corresponding dose–response	Models can be simple, and many off-the-shelf packages are available; captures exposure and response relationships	Powered by animal- and human-based studies; ignores detailed biological pathways, mechanism of action for therapies and biomarkers	Serum concentration of drugs and metabolites over time and dose–response curves
Virtual clinical trial	Varies – tends to be ODEs (Monolix, Phoenix, GastroPlus)	Drug development, drug repurposing and exploring combination therapies	Simulating monotherapy or combination therapy for dose regimens in specific virtual populations prior to preclinical *in vivo* experiments or clinical trials	Captures heterogeneity in both disease and response to treatment; captures multiple biomarkers and endpoints; explains variability across disease endpoints; can be used to explain combination therapies	Can be computationally expensive – requiring multiple simulations (one per treatment variation per member of virtual cohort); an extensive disease model requires relatively large amounts of preclinical and clinical data for calibration and validation	Preclinical data such as drug concentration and response curves; typical natural distribution of parameters of interest and distribution of biomarkers in population of interest; trial or population endpoints or outcomes
Clinical mathematical oncology	ODEs (Python, MATLAB, R)	Matching biomarkers to drug dosing schedule	Personalization of treatment (such as dosing schedule) based on biomarkers	Uses accessible clinical data to aid in clinical predictions and decisions	May provide relatively limited biological insight	Patient biomarkers, mutational status, treatment history, imaging studies and patient outcomes

ODEs, ordinary differential equations; PK, pharmacokinetics; PD, pharmacodynamics.

* MATLAB, Python and R are general scientific and computational programing platforms (MATLAB) and languages (Python and R), respectively. As such, they can be used for any category of mathematical model, especially with the wide variety of software packages available for them. We highlight them in the cases where there may not be model-specific software available. They are also often used for simple cases or when a specific use case is not covered by any other software.

### Model development and implementation

**Figure 2:**
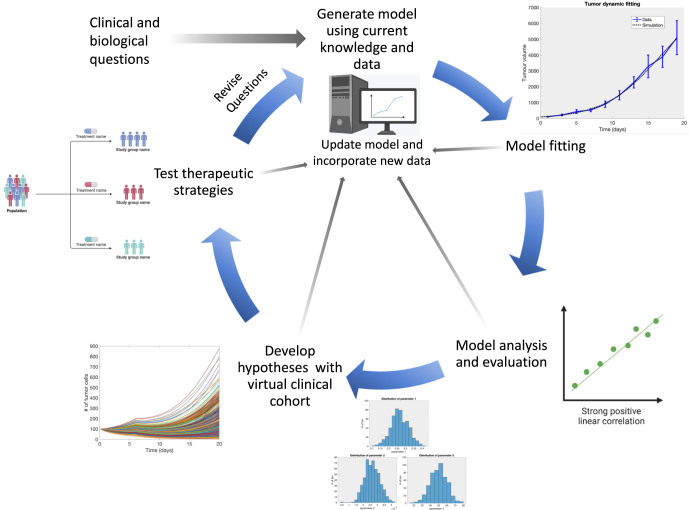
The multidisciplinary cycle of mathematical modeling spans the pathway from bench to bedside. We show an example model development cycle, in this case for a virtual clinical trial. Beginning with a clinical or biological question, a multidisciplinary team generates a mathematical model (for example, a systems biology or quantitative systems pharmacology model) informed with current knowledge and findings. The model is fit, or parameterized, with data, system behaviors analyzed, and results evaluated against additional datasets. Once the model is validated, a virtual patient cohort can be generated. Multiple treatment or experimental regimens can be tested computationally, producing testable hypotheses. Finally, the hypotheses can be tested in a clinical or experimental setting, with the cycle completing as questions are revised following testing of therapeutic strategies. At each step, the model can be updated with new findings and data, possibly starting the cycle over again. This process requires expertise from multiple disciplines and experiences. This includes patients who present with specific phenotypes, provide samples and inform the questions and trial designs; clinicians who provide specific clinical information and run clinical trials; bench scientists who perform preclinical research; and computational scientists who collaboratively produce the mathematical model. This cycle can help streamline preclinical and clinical research, all of which will be fed back to help with patient care. Example model outputs generated from MATLAB simulation code (with slight adaptations), were originally made available through the *Centre de recherches mathematiques (CRM) Computational Modeling of Cancer Biology and Treatments Workshop. *Original code available at https://github.com/mlcraig/ComputationalBiologyFittingTutorial. Aspects of figure adapted from Craig *et al.* 2023. Figure created in part with BioRender.com.

As illustrated in Fig. 2, motivated by a clinical need or biological question, the mathematical model is developed from the knowledge of the underlying biological system. This often takes the form of an interaction diagram. From there, the dynamics are translated to a mathematical representation, which can be encoded into one of several programs or general-purpose programming languages. See Table 2 for links to various software packages used in mathematical modeling. Then, a simulation can be run, feeding back into the original expectations and questions, leading to model refinement and the next step of the model development cycle. Once the model has attained sufficient initial accuracy, critical model parameters can be tuned by fitting to data. Finally, model predictions can be validated through existing data, new laboratory experiments or clinical trials.

### Systems biology and multiscale modeling

Systems biology is the practice of viewing biochemical interactions as a set of interconnected variables and processes, enabling the study of a whole system, rather than independent pathways. This includes developing the understanding of gene regulation, cell signaling networks, metabolic networks and the cellular information that produces a cellular state or behavior, such as proliferation or apoptosis. These phenomena are amenable to mathematical modeling and simulation. Techniques in this area include Boolean networks, ordinary differential equations (ODEs) and flux balance analysis (Ji *et al.* 2017, Voit 2018, Sahu *et al.* 2021, Rozum *et al.* 2023). See Table 2 for additional information. It is also possible to extend systems biology principles to cell-to-cell interactions and cell–environment interactions, leading to multicellular systems biology and multiscale modeling (Clarke *et al.* 2019, Metzcar *et al.* 2019). Multiscale modeling, or modeling that spans multiple times or spatial levels of organization, can represent phenomena such as the formation of spatial organization in a tissue or cancer, which includes both the microscale of cell behaviors and the macroscale of tissue structure (Clarke *et al.* 2019, Metzcar *et al.* 2019). Once important interactions are identified, such as the signaling pathways leading to aberrant proliferation, model structure and dynamics are set based on experimental data, hypotheses and the literature (Gómez Tejeda Zañudo *et al.* 2017, Rocha *et al.* 2021, Wu *et al.* 2022, Johnson *et al.* 2023). It is then possible to run the model, analyze its dynamics, make perturbations and confirm or refute the interactions and parameters of the model. As a result, new hypotheses can be formed and advance the experimental cycle (Fig. 1).

#### Systems biology examples

Systems biology models have been successfully used to advance precision medicine in endocrine cancers, including in breast and prostate cancers. For example, Tognetti and coworkers developed logic-based ODE models specific to breast cancer cell lines to predict drug sensitivity (Tognetti *et al.* 2021). They stimulated 67 cell lines with epidermal growth factor in the presence or absence of clinically utilized kinase inhibitors and assessed changes in 34 markers with single-cell mass cytometry to generate comprehensive signaling network models. They used the Genomics of Drug Sensitivity in Cancer dataset to validate their model, finding that it accurately predicted drug sensitivity. Their model identified features that predicted sensitivity or resistance to epidermal growth factor receptor and phosphoinositide 3-kinase (PI3K) inhibitors, as well as genomic variants that correlated with drug sensitivity predictions. Similar trends were also observed in patient-derived tumor xenograft models, demonstrating the possible use of cell signaling models to predict patient-specific responses to drug combinations.

Although there are multiple effective drug therapies available for estrogen receptor-positive (ER+) breast cancer, eventually, metastatic ER+ breast cancer develops drug resistance. To address this critical issue, a cell line-specific ODE model for ER+ and PI3K inhibitor-resistant breast cancer was developed to identify potential therapeutic drug combinations to increase drug sensitivity and overcome drug resistance (Gómez Tejeda Zañudo *et al.* 2021). This model predicted that the combination of the PI3K inhibitor alpelisib and a BCL-2 homology domain 3 (BH3) mimetic that targets anti-apoptotic proteins would result in a synergistic effect. Experimental results in MCF7 cells, a cell line model for ER+ PI3K-resistant breast cancer, validated the model prediction with the drug combination, leading to a greater increase in apoptosis compared to either drug individually.

Using a multiscale systems biology model in the context of triple-negative breast cancer (TNBC), Wu *et al.* (2022) integrated individual patients’ multiparametric MRI data with a 3D mathematical model of tumor cell movement, proliferation and therapy-induced cell death to predict patient-specific response. In essence, they created a digital twin to predict patients’ post-treatment status after the standard-of-care neoadjuvant systemic therapy (doxorubicin, cyclophosphamide and paclitaxel): pathological complete response (pCR) or non-pCR. Using the model prediction enables physicians to optimize individual treatment plans and select alternative therapies for patients unlikely to achieve pCR with neoadjuvant systemic therapy (Wu *et al.* 2022).

In prostate cancer, several major signaling pathways are dysregulated. In this context, Montagud *et al.* (2022) developed a Boolean network model capable of representing a range of these dysfunctions to guide personalized treatment. The authors created model variants using datasets from The Cancer Genome Atlas (TCGA), representing 488 patients and eight different prostate cancer cell lines. This approach enabled patient-/cell line-specific drug effect simulations and generated lists of potential patient-/cell line-specific treatments. The predicted drugs were experimentally validated in cell lines, suggesting that these models could successfully predict targeted therapies for patients (Montagud *et al.* 2022).

### Pharmacological modeling: physiology-based pharmacokinetic/pharmacodynamic and quantitative systems pharmacological modeling

Pharmacokinetic (PK) modeling and pharmacodynamic (PD) modeling describe serum levels of biologically active compounds and their effects on target tissues (Danhof *et al.* 2008, Rosenbaum 2016). More specifically, PK modeling describes the fluctuations of compound concentrations in the blood from the time of administration to its elimination from the body. By considering the body as a series of volumes that compounds pass through, a small set of ODEs are used to model blood concentration dynamics. PK models can be either single- or multicompartment models. A single-compartment model considers only blood concentrations to predict the concentration in other tissues. Multicompartment models account for compound exchange between different parts of the body (e.g., circulation and tissue) and are used as needed to better describe PK data. Finally, there are physiology-based PK (PBPK) models that model organ-level characteristics to closely follow compound concentrations and elimination from the body with the ability to include, for example, variations in drug metabolism and drug–drug interactions (Chen *et al.* 2012). In contrast, PD refers to the compound’s effect on the tissue rather than the effect of the body on the compound. The effect may be modeled as a simple Hill function parameterized by a cell viability or a drug–response curve. More complexity may be added when there is additional knowledge regarding the actual mechanism of action of a drug or drug class with various techniques used for this, such as systems or multicellular systems biology models. Extensions of PK/PD modeling include population PK, used to explore variation in PK across populations (Ette & Williams 2004), toxicity modeling, quantitative toxicity pharmacology and quantitative systems pharmacology (QSP). QSP models are more complex models that integrate systems biology knowledge (i.e., system immune response) and detailed pathways of drug mechanism of action (Chan *et al.* 2022). These are largely systems biology models simply used in the context of exploring drug development. Among other things, they can be used to explore multiple possible interventions that exploit the same mechanism of action or to study combination therapies (Gevertz & Kareva 2023).

#### Pharmacological modeling examples

Pharmacological models have also been successfully utilized in endocrine oncology research. In breast cancer research, Lai *et al.* (2019) developed a multiscale mathematical model that incorporates pharmacokinetics and pharmacodynamics with clinical data, including molecular profiling (tumor suppressor p53 status, vascular endothelial growth factor mRNA levels and hypoxia-inducible factor-1 α pathway deregulation score), histopathology and MRI, to predict treatment response in individual patients with HER2-negative breast cancer (Lai *et al.* 2019). They applied the model to patient data (*n* = 5) from a phase II clinical trial assessing the effectiveness of the anti-angiogenic drug bevacizumab, in combination with chemotherapeutic drugs fluorouracil, epirubicin and cyclophosphamide (FEC100). Fitting the model to each patient, they simulated observed patient outcomes, hypothesized how different patient outcomes were achieved and suggested alternative treatment regimes, showing that pharmacological modeling can facilitate precision medicine (Lai *et al.* 2019).

Pharmacological models have also been used to address cases of drug resistance or limited efficacy of a single therapy. Combination drug therapies that target multiple pathways involved in cell survival and proliferation can be highly effective when linked to patient-specific mutations and changes in gene expression. Tang *et al.* (2013) demonstrated that their network pharmacology model can be used for preclinical investigation to identify clinically relevant targets and therapies. To develop more effective therapies for patients with TNBC, their model integrates drug sensitivity with drug–target interactions to predict synergistic drug combinations. The MDA-MB-231 breast cancer cell line, which lacks estrogen receptors, progesterone receptors and human epidermal growth factor receptor 2 (HER2), similarly to TNBC, was used as a model system. Follow up dynamic modeling predicted synergy between the drug targets aurora kinase B (a mitotic regulator) and ZAK kinase (a p38 MAPK pathway regulator) and synergy between the drugs midostaurin (an aurora B inhibitor) and nilotinib (a ZAK inhibitor). *In vitro* experiments with both kinase inhibitors resulted in synergistic inhibition of cell growth and increased cytotoxicity (Tang *et al.* 2019).

In addition, these models can be used to predict treatment outcomes of patients. PRRT is used clinically to treat prostate cancer and neuroendocrine neoplasms. Patients with metastatic castration-resistant prostate cancer can receive [^177^Lu]-PMSA-617 (Sartor *et al.* 2021) and patients with SSTR-positive gastroenteropancreatic neuroendocrine cancer can receive [^177^Lu]-DOTATATE (Strosberg *et al.* 2017). Establishing a relationship between PRRT dosing and changes in tumor volume could improve therapy and treatment planning. Kletting *et al.* (2019) developed a mathematical model that incorporates PK, radiobiological effects (PD) and tumor volume sizes that predicted tumor volumes after PRRT in prostate cancer patients (Kletting *et al.* 2019). This model was based on PET/CT imaging data prior to the start of PRRT (*n* = 13 patients) and integrated with an established PK/PD model. Authors reported that their model was able to predict tumor volume in response to PRRT with a relative deviation of 1 ± 40%. The prediction accuracy was calculated by comparing the predicted tumor volume to the actual measured tumor volume 6 weeks after each patient received PRRT. This example highlights the utility of incorporating mathematical modeling to predict clinical responses.

### Virtual clinical trials

A virtual clinic trial is a computational technique that simulates aspects of a clinical trial or other population-based experiments (such as an animal-based trial). Virtual clinical trials enable researchers to test possible therapeutic interventions in a diverse population, including incorporating mechanistic knowledge such as the presence or absence of genetic mutations, without requiring any patient participants. They can inform expected outcomes of various dosing regimens in clinical trials by incorporating preclinical (*in vitro* and *in vivo*) data prior to beginning trials in patients, making the trial more likely to have a positive outcome (Pappalardo *et al.* 2019, Moingeon *et al.* 2023). Virtual clinical trials are based on a variety of techniques, including extensions of PK/PD modeling and systems biology modeling, and predict variability in outcomes (Craig *et al.* 2023). To produce the populations, the modeler induces variability in parameters anticipated to impact outcomes, such as drug clearance or drug–substrate interaction rates, and then accepts parameter sets that meet predetermined criteria. Each accepted parameter set joins the virtual population. Next, simulations, including perturbations in drug properties or dosing strategies, are run. The effects on the population emerge from the multitude of processes modeled, predicting the range of expected outcomes across multiple effects.

#### Virtual clinical trial examples

Virtual clinical trials provide a powerful platform to compare treatment strategies prior to clinical testing. For example, they have been applied to identify optimal drug treatment schedules to improve patient outcomes. Cheng *et al.* (2023) developed a virtual clinical trial for ER+ breast cancer patients using palbociclib (a cyclin-dependent kinase 4/6 inhibitor) with fulvestrant (an estrogen receptor antagonist) to explore alternatives to standard-of-care palbociclib dosing strategies (Cheng *et al.* 2023). The authors sought to determine whether alternative dosing strategies would be more effective, improve tolerability and overcome or delay resistance. They used *in vitro* drug synergy and cell cycle data to model the pharmacodynamics and integrated it with a pharmacokinetics model derived from clinical data. The virtual clinical trial results indicated that a continuous palbociclib treatment schedule is more effective than the standard pulsed-dose schedule, even if the cells acquire resistance to either palbociclib or fulvestrant. Their findings have been partially validated by existing clinical trials. Since their pharmacodynamic model was based on only one cell line, it is anticipated that the modeling of additional cell lines will enable further treatment schedule optimization.

Virtual trials facilitate the design of clinical trials, and in turn, ongoing clinical trials can also be used to modify a model and enhance its predictive power. Wang *et al.* (2020) developed a model to predict the anti-tumoral effects of monotherapies of nivolumab (an immunotherapy anti-PD-L1 drug), entinostat (a HDAC inhibitor with anti-proliferative activity) and/or ipilimumab (an immunotherapy anti-CTLA-4 drug) and their combination. By calibrating their model with data from the literature and clinical observations, their virtual clinical trial identified several potential predictive biomarkers that could be utilized to predict which patients will respond to a given therapy.

### Clinical mathematical oncology modeling

In addition to aiding the preclinical and clinical trial areas, mathematical modeling can be used in clinical decision making. The techniques and models previously described have potential to improve *in vitro* and *in vivo* preclinical experiments and the planning of clinical trials but may require complex data integration that takes time and modification to accurately predict outcomes. Furthermore, as useful as these models are in some circumstances, in the clinical area, there are often very limited data, at times leading to difficulty in precisely identifying parameters in complex models. In this case, simpler models that include fewer, more easily identified parameters are commonly utilized (Enderling 2022). They incorporate biological knowledge and offer immediate capabilities to guide decisions. These models tend to incorporate biomarker-specific data for the analysis being conducted. For example, levels of a specific blood-based biomarker or results from imaging data may be used to predict whether specific treatments will be efficacious, helping match patients with appropriate therapeutic regimes.

#### Clinical mathematical oncology modeling examples

Within prostate cancer, clinical mathematical oncology modeling has primarily used serum prostate-specific antigen (PSA) biomarker data as the basis for optimizing therapeutic approaches. PSA is an important clinical biomarker of prostate cancer used during both screening and treatment, with higher levels indicating increased disease burden. A mathematical model was developed that could predict the occurrence of relapse and if intermittent androgen suppression (IAS) could delay relapse (Hirata *et al.* 2010). The option to use IAS is an important clinical question in prostate cancer treatment, and mathematical modeling can provide parameters to guide optimal therapy. The model placed patients into three groups: predicted to respond to IAS for relapse prevention, delayed relapse from IAS and not predicted to respond. The mathematical model was built on access to clinical data, particularly data on the time course of serum PSA levels during therapy. This is an example of a feed-forward loop, in which clinical data are used to build a mathematical model that can then be used to refine clinical decision making. Clinical studies have also suggested that IAS has the benefits of maintaining androgen dependence of the tumor while increasing the patient’s quality of life.

Additional mathematical models of prostate cancer have also been developed to investigate the dynamics of androgen suppression while also studying the production of PSA (Portz *et al.* 2012). Their models utilize two cell populations: one that is androgen-dependent and another that is androgen-independent. The models’ goals were to develop a treatment strategy and to accurately predict the course of prostate cancer response per individual patient. Authors compared their models’ predictions to clinical data and saw a high level of accuracy.

In addition, a cell kinetics model was constructed to describe the evolution of prostate cancer from a local disease to systemic, androgen-independent disease (Dimonte 2010). In this model, the author incorporated a patient’s diagnostic data (e.g., PSA level, prostate volume, prostate cancer fraction and Gleason score) to predict survival time, with the goal of informing therapeutic decisions. The model included three different cell populations: local population sensitive to hormones, a regional population sensitive to hormones and a systemic, hormone-resistant population. There were three critical cell population levels that characterize the model’s dynamics: the cell level that initiates systemic cancer cell populations, the local tumor saturation level and the cell level likely to cause prostate cancer-specific death. The author reported that with validated parameters, this model could be used to estimate the median time to prostate cancer-specific death at an individual patient level.

Another effort, using the concept that continual treatment of androgen deprivation therapy can select resistant phenotypes and encourage treatment resistance (Zhang *et al.* 2017), modeled PSA dynamics to predict prostate cancer patient responses to IAS (Brady-Nicholls *et al.* 2020). The authors hypothesized that stem-like prostate cancer cells contribute to treatment failure due to self-renewing behavior and incorporated stem cell division patterns into their mathematical model to better predict treatment responses in patients. Calibrating a model of stem-like prostate cancer cell dynamics to available clinical trial data of PSA levels and patient outcomes, the model predicted the development of resistance with 89% accuracy. Overall, their results from modeling PSA dynamics to predict response provided clinically useful information regarding personalized treatment decisions using PSA values, evidence that treatment-resistant subpopulations may have affected outcomes in prior studies and that dynamics measured early in IAS therapy may help stratify which patients would benefit from continued IAS or the addition of other concurrent treatments, such as docetaxel.

An extension integrated metastatic burden and included PSA dynamics, stem-like prostate cancer cells and non-stem-like prostate cancer cells during adaptive treatment (Brady-Nicholls *et al.* 2021). The authors predicted patient response to adaptive therapy with 81% accuracy. Moreover, when authors simulated the addition of concurrent chemotherapy in patients predicted to progress, they found that the concurrent treatment had a 1–11% reduction in the probability of progression compared to patients treated with adaptive therapy alone. The work highlights a direct connection between mathematical modeling and a relevant clinical need to improve patient outcomes.

## Current state of modeling in NETs

Although mathematical modeling has been utilized in endocrine cancers, to date, few mechanistic models have been developed for NETs. For example, to aid in the planning of preclinical *in vivo* mouse experiments, Walls *et al.* (2012) generated a simple ODE to predict the growth rate of pituitary tumors and pancreatic NETs in *MEN1* knockout mice (Walls *et al.* 2012). The model analysis provided insights into the use of these mice and created the opportunity to model the effects of different therapeutic agents.

Another research effort includes a game theory model of cooperative cell populations (Archetti *et al.* 2015). The authors generated a model to examine cell population dynamics under the influence of insulin-like growth factor-II (IGF-II) using data from insulin-expressing pancreatic NET (insulinoma) cell lines derived from the Rip1Tag2 mice with either wild-type or IGF-II deletion. Insulinoma cells that do not produce IGF-II grow slowly in pure cultures but have a growth advantage in mixed cultures. The model investigates the balance between cells that secrete or do not secrete IGF-II. They note that treatment of insulinomas with growth factor-targeting drugs may result in an initial reduction in tumor growth but could be followed by a new population equilibrium that ultimately promotes tumor growth and may explain drug resistance in patients. This model predicts the time this would occur, generating a testable hypothesis that could aid in planning schedules for *in vivo* experiments and potentially clinical trials.

Werle and coworkers developed a systems biology approach to study mechanisms of drug sensitivity, resistance and phenotypes using a Boolean network approach (Werle *et al.* 2023). Their model included 56 nodes (representing genes or proteins) and 195 interactions, capturing aspects of the MAPK, angiogenesis, PI3K/AKT, mTORC, cell adhesion and cell cycle pathways leading to the phenotypes of quiescence, detachment, G0-alert and angiogenesis. They performed virtual gene knockouts, validating their model through comparison to RNA-based phenotyping and recapitulating a range of simulated phenotypes matching established knowledge of pancreatic NETs (heterogeneity and relative indolence). Finally, using the validated model, they explored the efficacy of and resistance to mTORC1 inhibition, with results suggesting that patients with MEN1 loss are more likely to benefit (experience control to a quiescent phenotype) compared to patients with tuberous sclerosis (TSC) or DAXX loss. Their model further predicted that mTORC1 and mTORC2 inhibition may produce an even better response.

A physiology-based pharmacological model was developed to predict the kinetics and biodistribution parameters of [^177^Lu]-DOTATATE (Jitsinchayakul *et al.* 2022). The model predicts the concentration of [^177^Lu]-DOTATATE throughout the body to calculate the radiation exposure to each organ and optimize possible toxicity in organs at risk (e.g., the kidney and the liver) versus exposure to the tumor. The model is based primarily on mouse data. However, with additional development, it could provide valuable insights into [^177^Lu]-DOTATATE dosing for individual NET patients.

Finally, current efforts include models that can aid in clinical practice and in NET research. For example, one model used a regression growth model and computed tomography imaging data from lanreotide depot/autogel- and placebo-treated NET patients from the CLARINET trial to predict the expected growth rate and doubling time of a patient’s tumor in the early course of treatment (Dromain *et al.* 2021). This model could therefore guide a clinician in treatment decisions based on whether it appears that a patient is responding or not.

## Practical notes on model development and implementation

There are numerous paths for developing and implementing mechanistic models in the computational setting. Table 2 provides a variety of details, including links to useful modeling software, example use cases and typical kinds of data used in model development and validation. Typically, the resulting computational implementations can generally be run on standard, consumer-grade hardware, such as a typical desktop or laptop. At times, high-performance computing may be required, for example for an in-depth parameter exploration of a large agent-based model. All modeling efforts require data processing at some point. This includes cleaning data, converting to formats for fitting algorithms, data visualization and, finally, the matching of the data to the computational model (fitting). There are a range of options for this, and it would typically be done in the framework the model is written in, such as Python, MATLAB or R. See Simpson and Baker for examples of data fitting in Python (Simpson & Baker 2024). Memory requirements for integrating models with datasets may be low, again, with high-performance computing indicated for very large datasets.

Researchers use a range of data types to develop and validate mechanistic models. Once a model is generated based on existing knowledge, the data serve first to specify a model to a particular system and then, with independent data, to validate the implemented approximation of the biomedical reality. Thus, unlike in data-driven modeling, which often benefits from large amounts of data, mechanistic modeling benefits from focused datasets, which may be smaller, but capture the dynamics or changes in the system under study (Metzcar *et al.* 2024). Examples of data types used in modeling include omics (including spatial), brightfield and confocal microscopy images, flow cytometry, pharmacokinetics, patient data such as treatment history and tumor mutational status, and clinical imaging. See Table 2 for additional details.

## Conclusions and future perspectives

Mathematical modeling is an established area of research that has the potential to complement and improve preclinical and translational studies, clinical trial design and even clinical practice. As seen in the reviewed literature, it is already in use in breast and prostate cancer research. While there are comparatively few studies in NETs, scoping out the requirements for mathematical modeling, we see there is great potential to transfer those successes for use in NETs and RETSs to advance disease understanding, streamline preclinical biological experiments and enhance patient and therapy selection for clinical trials and informing clinical decisions – all needs for these uncommon or rare diseases.

As a concrete example, more than 20 mutated genes have been implicated in the oncogenesis of NETs in addition to a host of epigenetic alterations, all impacting multiple biological pathways (Öberg 2013, Di Domenico *et al.* 2017, Scarpa *et al.* 2017). This mutational landscape plays a significant role in determining treatment responses. Using network models that can account for the consequences of these differences in genetics could help streamline biological experiments. To aid in identifying a suitable drug for the treatment of pancreatic NETs with mutations in *MEN1* and *DAXX*, one could use a systems biology model, perhaps through extension of the modeling work of Werle *et al.* (2023), to identify drugs that are most likely to be effective. This may avoid or limit cell line screening and enable going directly to *in vivo* and *ex vivo* validation. The use of a computational model would spare both cost and materials from valuable biological resources. In addition, the computational model could be used to identify drug combinations, a process that is otherwise very resource intensive. Streamlining preclinical studies could expedite the time to undertake clinical trials, enhancing translational studies.

The mathematical modeling techniques described above also provide an opportunity to undertake virtual clinical trials to make the most of the limited number of accessible NET patients. This is particularly prevalent in syndromic NET patients, such as those with multiple endocrine neoplasia type 1 (MEN1), who carry hereditary heterozygous mutations in the *MEN1* gene. These patients develop NETs affecting multiple organs. Due to the complex nature of their disease, these patients are often excluded from clinical trials, meaning access to data and patient material is limited, and treatments are based on results from trials on single-site sporadic tumors. Being able to undertake clinical trials based on mathematical models would not only provide an opportunity to design appropriate trials for MEN1 and, more generally, RETS patients to enter, but also help translate experimental approaches that are already in use for other NET patients.

Additional areas of interest in the field include immunotherapies and radiopharmaceutical treatments – both complex treatments that include cell–cell interactions and/or difficult-to-predict responses. Mathematical modeling, with its ability to include dynamics across spatial and time domains, is well poised to aid in understanding the effects of multiple immune species and therapeutic agents interacting in potential immunotherapy combinations (Takkenkamp *et al.* 2020, Popa Ilie & Georgescu 2023, García-Torralba *et al.* 2024) and biomarkers of efficacy and toxicity in the low-dose, long-term radiation of PRRT (Bodei *et al.* 2020, Hope *et al.* 2022).

Mathematical modeling is not without its challenges. To develop these models, an interdisciplinary approach is required whereby clinicians, laboratory-based bench scientists and mathematical modelers work together to integrate knowledge of the disease and treatments and patient and preclinical data to ultimately lead to improved therapeutic options (Fig. 2). Of particular importance is the generation and sharing of preclinical pharmacological, omics (for example DNA sequencing and RNA sequencing), tumor microenvironment and patient outcome data, ideally with multiple time points across experiments. There are available omics datasets, for example, RNA sequencing of GEP-NETs (GSE98894), pNET-specific RNA sequencing (GSE73338) and pNET genotyping and expression data (GSE117851), the latter two being used in the study by Werle *et al.* (2023). The study by Alcala and coworkers made public omics data obtained from patient-derived organoids across a range of NETs (Alcala *et al.* 2024). Additional datasets for a variety of NET subtypes can be found in repositories such as GEO Datasets, the European Genome-Phenome Archive and ProteomeXchange. In addition, there are studies of the tumor microenvironment (including the immune environment) that can serve as a valuable base for model building and validation (Cives *et al.* 2019, Takkenkamp *et al.* 2020, Niedra *et al.* 2025). While there is information on NETs and RETSs, as evidenced by the available data, there remains a gap in knowledge in these understudied diseases. Mathematical modeling, as part of the research cycle, provides an opportunity to increase the efficiency of preclinical and clinical research and help advance NET patient outcomes. Then, as more data are generated, mathematical and computational models can be improved, which can then further additional experiments in a positive-feedback loop.

Just as mathematical modeling has become relatively common in other areas of cancer study, we hope this work, which highlights the utility of mathematical modeling in cancer studies, encourages dialog and collaborative research among the scientific, clinical and computational modeling communities. In laying out the requirements for mathematical modeling, we hope the design of new experiments includes gathering the focused data that will benefit mathematical modeling, which in turn will benefit future experiments. Given this opportunity to exponentially accelerate experiments, we are excited to see the results of incorporating mathematical modeling into future NET studies.

## Declaration of interest

The authors declare that there is no conflict of interest that could be perceived as prejudicing the impartiality of the work.

## Funding

JM was partially supported by the National Science Foundationhttps://doi.org/10.13039/100000001 NRT (Grant no. 1735095).

## Author contributions statement

JM, RG, YW, KMB and KEL all equally contributed to literature searches, manuscript writing and editing.
